# Cytokine Milieu Relevant to Atopic Dermatitis Enhances Epidermal Trypsin‐Like Serine Protease Activity, Which Is Pharmacologically Suppressed in Human Keratinocytes

**DOI:** 10.1111/1346-8138.70160

**Published:** 2026-01-27

**Authors:** Shiori Onishi, Yohei Yasutomi, Onon Tsedendorj, Shin Morizane

**Affiliations:** ^1^ Department of Dermatology Okayama University Graduate School of Medicine, Dentistry and Pharmaceutical Sciences Japan

**Keywords:** atopic dermatitis, epidermal keratinocytes, kallikrein‐related peptidases, trypsin‐like serine protease activity

Dear Editor,

1

Atopic dermatitis (AD) is a chronic inflammatory skin disease characterized by epidermal barrier dysfunction, type 2–dominant inflammation, and pruritus, which together contribute to disease development and persistence [1]. Aberrant activation of epidermal serine proteases has attracted attention as an important factor linking inflammation to barrier disruption [2]. In human epidermal keratinocytes, multiple kallikrein‐related peptidases (KLKs) are expressed and regulate desquamation by cleaving corneodesmosomal components, including desmoglein‐1 (DSG1), desmocollin‐1 (DSC1), and corneodesmosin (CDSN) [2]. Excessive protease activity, however, compromises barrier integrity and may amplify cutaneous inflammation [2]. The pathological relevance of KLK dysregulation has been demonstrated in experimental models. Transgenic mice overexpressing human KLK5 (trypsin‐like serine protease) exhibit allergic dermatitis accompanied by severe inflammation and pruritus [3]. Transgenic mice overexpressing human KLK7 (chymotrypsin‐like serine protease) develop chronic pruritic dermatitis similar to AD [4]. Clinical studies have further shown that both chymotrypsin‐like and trypsin‐like serine protease activities are elevated in lesional AD skin, supporting the clinical relevance of protease dysregulation [5].

We previously reported that Th2 cytokines such as interleukin (IL)‐4 and IL‐13 enhance chymotrypsin‐like serine protease activity in keratinocytes, whereas trypsin‐like activity was not sufficiently induced by these cytokines alone [6]. These observations suggested that additional inflammatory signals are required to recapitulate the protease activation observed in AD lesions. Given that AD skin is characterized by the simultaneous upregulation of multiple cytokines derived from Th2, Th17, Th22, and other immune pathways, we hypothesized that a composite cytokine milieu, rather than individual cytokines, is critical for inducing epidermal trypsin‐like serine protease activity.

To address this issue, normal human epidermal keratinocytes (NHEKs, obtained from Cascade Biologics/Invitrogen) were simultaneously stimulated with six cytokines known to be upregulated in AD lesions (IL‐4, IL‐13, IL‐17A, IL‐22, IL‐31, and IFN‐γ) in the presence of calcium (2 mM). After 96 h, trypsin‐like serine protease activity in the culture supernatant was significantly enhanced compared with unstimulated controls (Figure 1A). In contrast, stimulation with each cytokine alone induced only minimal changes in activity.

**FIGURE 1:**
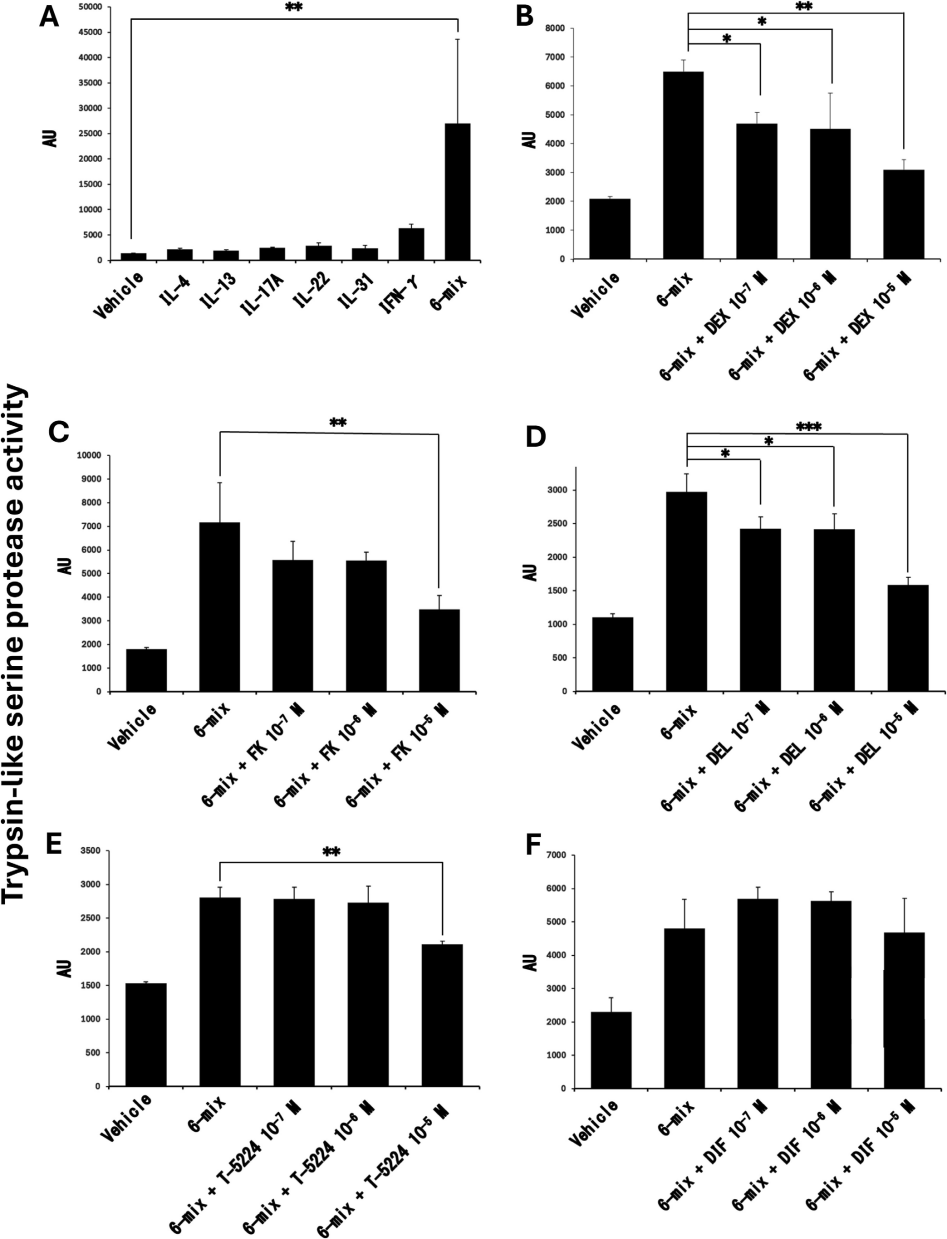
Cytokine‐driven enhancement and AD therapeutic suppression of epidermal trypsin‐like serine protease activity in NHEKs. (A–F) NHEKs were stimulated with IL‐4, IL‐13, IL‐17A, IL‐22, IL‐31, IFN‐γ (50 ng/mL each), DEX, FK, DEL, T‐5224, and/or DIF in the presence of 2 mM Ca^2+^ for 96 h. Trypsin‐like serine protease activity in the cultured media was measured using Boc‐Phe‐Ser‐Arg‐MCA as a specific substrate. *p < 0.05, **p < 0.01, ***p < 0.001. Data are mean ± SEM of triplicate samples and are representative of three independent experiments. AU, arbitrary unit; DEX, dexamethasone; FK, FK506; DEL, delgocitinib; DIF, difamilast.

We next examined the effects of topical agents commonly used for AD treatment on this cytokine‐induced protease activation. Dexamethasone, tacrolimus, and the topical Janus kinase inhibitor delgocitinib significantly attenuated the enhanced trypsin‐like serine protease activity in a concentration‐dependent manner (Figure 1B–D). In addition, the activator protein‐1 inhibitor T‐5224, a candidate topical anti‐inflammatory agent, also significantly suppressed protease activity (Figure 1E) [7]. In contrast, the phosphodiesterase 4 inhibitor difamilast did not markedly affect trypsin‐like serine protease activity under these conditions (Figure 1F). Although the magnitude of enhancement varied among experiments, the direction of change was consistent across independent assays.

This study is limited to an in vitro keratinocyte system and does not identify the specific proteases or intracellular signaling pathways responsible for the observed increase in trypsin‐like serine protease activity. Nevertheless, our findings demonstrate that a cytokine milieu relevant to AD is sufficient to enhance epidermal trypsin‐like serine protease activity and that this excessive activity can be pharmacologically suppressed by several topical agents. These observations provide pathophysiologically relevant insights into epidermal protease dysregulation under inflammatory cytokine environments and may help guide therapeutic strategies targeting barrier dysfunction in inflammatory skin diseases.

## Funding

This work was supported by the Japan Society for the Promotion of Science, JP23K07766.

## Disclosure

Shin Morizane is an Editorial Board member of Journal of Dermatology and a co‐author of this article. To minimize bias, they were excluded from all editorial decision‐making related to the acceptance of this article for publication.

## Conflicts of Interest

S.M. has received research funding from Sun Pharma Co. Ltd. and Maruho Co. Ltd., and honoraria from Eli Lilly Japan K.K., UCB Japan Co. Ltd., AbbVie GK, Pfizer Japan Inc., Torii Pharmaceutical Co. Ltd., Sanofi, K.K. Boehringer Ingelheim Co. Ltd., and Maruho Co. Ltd. The other authors declare no conflicts of interest.

## Data Availability

Raw data were generated at the Department of Dermatology, Okayama University Graduate School of Medicine, Dentistry, and Pharmaceutical Sciences. Derived data supporting the findings of this study are available from the corresponding author S.M. on request.
